# Optimising Calorie Intake for People With Amyotrophic Lateral Sclerosis: A Process Evaluation of a Complex Behaviour Change Intervention

**DOI:** 10.1111/hex.70417

**Published:** 2025-09-01

**Authors:** Katie Hullock, Alicia O'Cathain, Fiona Sampson, Jonathan Woodward, Elizabeth Coates, Theocharis Stavroulakis, Sean M. White, David White, Paul Norman, Christopher McDermott

**Affiliations:** ^1^ Sheffield Clinical Trials Research Unit (CTRU), Sheffield Centre for Health and Related Research The University of Sheffield Sheffield UK; ^2^ Sheffield Centre of Health and Related Research The University of Sheffield Sheffield UK; ^3^ Division of Neuroscience, School of Medicine and Population Health, Sheffield Institute for Translational Neuroscience The University of Sheffield Sheffield UK; ^4^ Department of Psychology The University of Sheffield Sheffield UK

**Keywords:** amyotrophic lateral sclerosis, energy intake, qualitative research

## Abstract

**Objective:**

To explore intervention fidelity and experiences of using a new intervention designed to optimise calorie intake in people with amyotrophic lateral sclerosis (ALS).

**Methods:**

A mixed‐methods process evaluation was conducted alongside an ongoing randomised controlled trial across 15 ALS specialist centres in the United Kingdom. Data collection included 146 healthcare professional‐completed fidelity checklists, audio recordings of 5 intervention sessions, and qualitative interviews with 32 healthcare professionals, patients and informal caregivers.

**Results:**

Intervention fidelity was high (88%: 1059/1204 items completed by healthcare professionals). Healthcare professionals, patients and informal caregivers within the sample recognised the intervention's value and engaged with it. Patients were motivated to use the intervention because they believed it could slow disease progression, and it gave them a sense of control. Despite challenges with the intervention, including patient concerns about weight gain and physical limitations in food preparation and consumption, patients in this sample remained committed to using the intervention. However, healthcare professionals suggested that these challenges may have negatively influenced trial recruitment and retention. Caregivers played a crucial role in supporting patients emotionally and physically, helping them to adhere to the intervention.

**Conclusions:**

The intervention was feasible to implement and was delivered with fidelity. While patient engagement in this sample was strong, the intervention usability may be time‐limited as physical function declines. Therefore, the intervention may be best suited for those with slower‐progressing ALS who can manage the intervention and dietary changes. Moving forward, continued evaluation is needed to assess effectiveness and explore subgroup differences based on ALS type (slow vs. fast progressing).

**PPIE Contribution:**

PPI was integral to the process evaluation. PPI members reviewed key study documents, including the Participant Information Sheet and consent forms, leading to participant materials that were clear and easily understood. They also participated in developing the intervention.

**Trial Registration:**

IRAS ID 275949, ISRCTN30588041.

## 1 Introduction

Amyotrophic lateral sclerosis (ALS) is a neurodegenerative disease characterised by the loss of motor neurons, which causes progressive paralysis and eventually death [1]. It is commonly known as motor neuron disease. There is no cure for ALS, so treatment focuses on slowing progression and managing symptoms [2]. Recent research has focused on the role of weight, nutrition and calorie intake in ALS. This is because people with ALS often experience increased resting energy expenditure and difficulties maintaining adequate nutritional intake. This leads to progressive weight loss, with hypermetabolism and suboptimal nutritional intake making weight loss worse [3]. While some studies indicate that higher caloric intake might stabilise weight and potentially improve survival rates, the overall evidence is mixed and further research is required to measure potential benefits [3, 4].

Systematic reviews with meta‐analysis have shown positive results for interventions aiming to increase calorie intake. In one review, a high‐calorie diet improved body weight without increasing adverse events, although it showed no effect on longer‐term outcomes such as survival rates and quality of life [5]. Another review of high‐calorie supplementation concluded that it was safe, well‐tolerated and generally beneficial but showed no change for some outcomes [6]. Authors of these reviews concluded that there is a need to undertake further high‐quality clinical trials to identify the effect of interventions, particularly because concordance is either low or unknown, which is likely to limit their effectiveness [5, 6]. There is also a need to undertake research to understand how nutritional management is structured and managed [7].

A digital behaviour change nutrition intervention was developed to optimise weight in people with ALS, aiming to help them meet their estimated calorie requirements. It was informed by systematic reviews, surveys and qualitative interviews with people with ALS, their informal caregivers and healthcare professionals [8, 9]. The COM‐B framework (Capability, Opportunity, Motivation) underpinned the intervention design [10]. Research highlighted the need to tailor the intervention to individual symptoms and preferences and to explain the rationale for dietary changes, especially for those who previously followed healthy eating guidance [11]. The intervention was a complex intervention in that it comprised multiple components delivered over a 12‐month period [12]. It was delivered via a website/online portal, supported by a healthcare professional. The site explained how calorie targets were set, detailed the intervention's rationale, provided food diary tools, feedback on calorie intake and weight, personalised plans, and practical resources. A digital format was chosen over a booklet to enable individualised feedback and easier access for people with ALS, who often already use technology.

Healthcare professionals delivering the intervention, either specialist dietitians or research nurses, introduced the website, set targets, educated patients on increasing intake, and provided oral nutritional supplements if needed. Follow‐up visits occurred at months 1, 3, 6, 9 and 12 to adjust targets. Though initially planned as in‐person, delivery shifted to remote (video/phone) due to Covid‐19, which proved acceptable and more convenient for patients. Professionals received training and monthly group supervision with the research team.

The intervention was developed for patients with symptom onset within the past 2 years, who were more likely to be physically able to use the website. Results are pending.

While randomised controlled trials measure effectiveness, process evaluations can explain the trial results by assessing the fidelity of delivering the intervention, exploring the relationship between context and outcomes, identifying mechanisms of impact, and exploring the feasibility, acceptability and implementation of the intervention. The process evaluation reported here addressed the following research question: Is a new intervention developed to optimise calorie intake for people with ALS delivered with fidelity, and feasible and acceptable to healthcare professionals, patients and informal caregivers?

## 2 Methods

### 2.1 Design

The design was a mixed‐methods process evaluation, following published guidance [12, 13, 14]. It consisted of three concurrent components: a fidelity assessment of delivery of the intervention, that is, whether the intervention was implemented as planned; qualitative interviews to explore the experiences of healthcare professionals delivering the new intervention within the trial; and qualitative interviews with people with ALS receiving the intervention and their informal caregivers.

The process evaluation took place alongside a randomised controlled trial that planned to recruit 259 people with ALS over 23 months in up to 20 specialist centres in the United Kingdom. By November 2023, after 29 months, the trial had recruited 75 participants in 15 centres. The slow trial recruitment was largely due to patients wanting to participate in drug trials and not wanting to participate in two trials simultaneously, and the Covid‐19 pandemic disrupting research processes in hospitals in the United Kingdom. 28 of the 75 participants withdrew from the trial, leaving 22 in the intervention arm. Reasons for withdrawal included finding the trial too demanding or time‐consuming, recruitment to drug trials, being too unwell to proceed, and/or finding participation in research burdensome.

### 2.2 Sampling

The process evaluation started a few months after the trial began. It was stopped earlier than planned (in November 2023), partly due to the inefficiency of continuing the process evaluation when the trial was recruiting at a slower‐than‐expected rate, and partly due to data saturation for the patient and carer interviews. In terms of data saturation, during data collection, the qualitative researcher noted that they were hearing the same issues in the last few patient and carer interviews. There was no option to diversify the sample at that stage because of the slow recruitment in the trial, so data collection was stopped. The inclusion criteria for the trial at the time of the process evaluation were that people with ALS were aged over 18 and within 2 years of symptoms of ALS developing. Details of the inclusion criteria are published elsewhere [11]. Because trial recruitment was much slower than expected, all participants in the intervention arm of the trial were approached for qualitative interviews, rather than undertaking purposive sampling.

For fidelity, the focus was treatment delivery [11]. To assess this, healthcare professionals delivering the intervention completed a checklist electronically to identify what they covered in each of the six intervention visits. All checklists completed up to November 2023 were included in the analysis. In addition, healthcare professionals delivering the intervention were asked to audio record an intervention visit using an encrypted recorder and send this recording to the research team for analysis.

For the qualitative interviews with healthcare professionals, the aim was to interview 20 people who have experience in delivering the intervention. Written informed consent was sought before an interview. For the qualitative interviews with patients in the intervention arm of the trial, and their informal caregivers, the aim was to interview 20 patients when they had used the intervention for between 1 and 3 months to get their views about initial use of the intervention, and 20 when they had used the intervention for 6–12 months to get their views of sustained use of the intervention. The aim was to approach some patients at both time periods to consider changes in their views over time.

### 2.3 Data Collection

Semi‐structured interviews were undertaken with healthcare professionals by video call using a topic guide covering their role in the trial, and their experiences of training in, and delivering, the intervention. Interviews lasted between 18 and 39 min. Semi‐structured interviews were undertaken with patients and informal caregivers either by telephone or video call using a topic guide covering their diagnosis, symptoms, approach to food and nutrition, intervention training received, and their experience using the intervention. Interviews took place between March 2022 and November 2023 and lasted between 20 and 84 min. Patients and their informal caregivers were approached for separate interviews, but they sometimes chose to participate in an interview together. Interviews were recorded and transcribed verbatim by a transcribing service, and transcripts were anonymised.

### 2.4 Interviewing Patients With ALS

ALS may impact people's ability to communicate. To address this, the qualitative interviewer sent them a short Communication Support Plan before arranging the interview to identify what help they might need. Most patients were happy to participate without support, except one patient who preferred to send an email response to the topic guide questions because they were unable to speak. During the interviews, some patients offered short answers, which may have been related to the effort required to speak.

### 2.5 Analysis

The healthcare professional‐completed fidelity checklist data were exported from the central trial database and analysed using Microsoft Excel. J.W. calculated counts and percentages for each checklist item for each visit type. Each item on each checklist was given equal weighting (i.e., if 7 of 10 items on a checklist were delivered, then that intervention visit had 70% fidelity). Missing and not applicable responses were excluded (i.e., on a 10‐item list, 5 ‘yes’, 3 ‘no’ and 2 blank would have a fidelity of 5/8 or 62.5%). The small number of missing responses was excluded because they appeared to be missing at random. The overall fidelity was calculated using the number of fidelity responses of ‘Yes’ across all checklists divided by the sum of the number of fidelity responses of ‘Yes’ and ‘No’. For the recorded intervention sessions, a researcher completed a fidelity checklist comprising 10 items.

K.T. used the ‘framework approach’ to analyse the interviews [15]. This was selected because the different aspects of a process evaluation offered an a priori framework for analysis. K.T. undertook the following steps of the framework approach. First, K.T. read the transcripts for familiarisation. Second, K.T. created a coding framework based on the aims of a process evaluation (fidelity, implementation, mechanisms of action, context, acceptability and feasibility) and further themes identified inductively from reading the transcripts. Third, K.T. applied this coding framework to all the transcripts. The COM‐B model had been used to develop an intervention to change eating behaviour [10]. This framework of theories comprises Capability (including knowledge, skills, decision processes and habits), Opportunity (including environmental context and social influences), and Motivation (including confidence, identity, beliefs about consequences, emotions, goals, intentions, reinforcing behaviour and optimism/pessimism) in influencing behaviour [10]. As the analysis progressed, K.T. and A.O.C. identified a close fit between the COM‐B model and developing themes and used it to structure the analysis. Quotes are used to illustrate findings. Labels P, C, P&C and HP are used to identify patients, caregivers, joint patient/caregiver and healthcare professionals. Labels of ‘early’ and ‘late’ were used to indicate interviews undertaken at the early stages of intervention use and later stages of intervention use.

## 3 Results

### 3.1 Description of Fidelity Samples

A total of 146 health professional‐completed fidelity checklists were available for the six visits for each participant in the intervention arm: 1 week visit (*n* = 37 checklists); 1 month visit (*n* = 32); and 3 month (*n* = 25), 6 month (*n* = 21), 9 month (*n* = 16), and 12 month (*n* = 15) visits. Two of the 1‐week checklists were subsequently excluded because the participant withdrew from the trial during the session, leaving 144 checklists for analysis. Five intervention visits were audio recorded for the research team to assess fidelity. They were from four different centres, with four different healthcare professionals delivering the intervention, and covered visits at 1 month, 6 months, 9 months and 12 months.

### 3.2 Description of Interview Participants

Eight interviews were undertaken with healthcare professionals delivering the intervention in six centres: six dietitians, one research nurse and one clinical research coordinator. The centres were from a wide geographical spread across England and Scotland and included both large teaching hospitals in the form of regional hubs and smaller district general hospitals. 19 healthcare professionals were approached in 9 centres for interview. Most did not respond to the invitation or reminder, and one reported having no one in the intervention arm of the trial, resulting in eight interviews.

25 interviews were undertaken with patients, caregivers or a combination of the two across six centres. Overall, 27 individuals took part in these interviews. Interviews took place early in the process of using the intervention (*n* = 12 interviewed between 1 and 4 months) and others later in the process (*n* = 8 interviewed between 5 and 12 months, *n* = 3 interviewed after 12 months). Characteristics of participants are described in Table 1. Participants had a mean of 6 months between their week 1 intervention visit and interview. Most had clinically definite ALS, and most were in slow decline (see footnote of Table 1). Caregivers were the partners or spouses; in one interview, two caregivers were present. Interviews were undertaken on two occasions with three patients/carers.

**Table 1:** Characteristics of patients in the interview study.

Participant ID	Time lapsed since patient diagnosis	El‐Escorial classification for patient	ALSFRS decline category^a^	Centre ID	Early in use of intervention (< 5 months) (E)/Late (L) (5 months onwards)
C1	12 months	Clinically definite ALS	Slow decline	5	L
C2	9 months	Clinically probable ALS	Slow decline	1	L
C3	3 months	Lab supported ALS	Slow decline	1	E
C4	17 months	Clinically definite ALS	Slow decline	2	L
C5	8 months	Clinically probable ALS	Slow decline	1	L
P&C1	2 months	Lab supported ALS	Slow decline	1	E
P2	9 months	Progressive muscular atrophy	Fast decline	4	L
P&C3 L P&C3 L	5 months 14 months	Clinically definite ALS	Slow decline	4	L, L
P4	Unknown	Unknown	Unknown	Unknown	Unknown
P&C5	11 months	Clinically definite ALS	Fast decline	4	L
P6 E P6 L	3 months 11 months	Clinically definite ALS	Slow decline	2	E, L
P7	10 months	Clinically probable ALS	Slow decline	6	L
P8	7 months	Clinically definite ALS	Slow decline	3	L
P&C9	3 months	Clinically probable ALS	Slow decline	1	E
P10	2 months	Clinically definite ALS	Slow decline	5	E
P11	2 months	Clinically definite ALS	Slow decline	2	E
P12	2 months	Clinically definite ALS	Slow decline	5	E
P13	2 months	Lab‐supported ALS	Slow decline	1	E
P14 E P14 L	1 month 5 months	Clinically definite ALS	Slow decline	2	E, L
P15	3 months	Clinically definite ALS	Slow decline	2	E
P16	1 month	Clinically definite ALS	Slow decline	3	E
P17	4 months	Clinically definite ALS	Slow decline	5	E

^a^ Based on ALSFRSr, a measure of functionality on four domains with scores ranging from 0 to 48. Rate of change in this score was measured each month, with higher values indicating faster decline. Decline above 0.9 on average was labelled as ‘fast decline’.

### 3.3 Fidelity—The Intervention Was Used as Planned

1256 items should have been completed by healthcare professionals. 13 were missing, and 40 were ticked as not applicable, leaving 1203 items. The average fidelity for the healthcare professional‐completed fidelity checklists was 88% (1059/1203). In conducting the analysis, it became clear that one of the fidelity fields had a significantly lower score than the others (‘Has the participant seen a dietitian since their last visit?’), with only 27% (38/143) positive/affirmative responses. In two of the audio‐recorded intervention visits, healthcare professionals asked the patient whether they had seen a dietitian since the last visit, but when the participant said ‘No’, the fidelity checklist was marked as ‘No’ rather than ‘Yes’ to indicate that they had asked the patient about this. The healthcare professional‐completed fidelity increased to 96% (1021/1060) when discounting the dietitian question. The researcher‐completed fidelity checklists, based on audio recordings of 5 intervention visits, also showed high fidelity, with 83% (33/40) average fidelity when including the dietitian question and 86% (30/35) average fidelity when the dietitian question was discounted. The researcher‐completed fidelity checklists were compared with the healthcare professional‐completed checklists for the same visits. There was a high agreement between the two, with the researcher and health professional agreeing on 84% of fields (32/38) when the dietitian question was included, and 91% (30/33) of fields when the dietitian field was discounted. Two fields were not compared because the health professional ticked ‘N/A’, and the researcher did not have the clinical experience to assess whether it was not applicable.

During the interviews, the healthcare professionals described how they appreciated practical assistance from the research team when conducting intervention sessions. They experienced some tension between fidelity and tailoring the intervention to individuals. Healthcare professionals described the complexity of the health issues faced by some patients, for example, the presence of comorbidities and ALS symptomatology, which might impact diet. They felt that a personalised approach to the intervention was vital: ‘*It's always based on the patients and you have to tailor it to them’* (HP6). The complexity of patients' health issues reinforced the need for a personalised approach.

Patients and informal caregivers generally understood the intervention, with most patients expressing a willingness to follow the dietary advice. However, difficulties arose with consuming oral nutritional supplements, where caregiver encouragement was sometimes necessary, but not always effective.

### 3.4 Overview of Themes

When trying to understand how healthcare professionals, patients and their caregivers reacted to the intervention, we identified nine sub‐themes related to the COM‐B framework of theories: three sub‐themes related to capability, two to opportunity and four to motivation [10].

### 3.5 Capability: Patients' Ability to Physically Use the Intervention

The COM‐B framework describes capability as having the psychological and physical abilities to perform the behaviour, including knowledge skills and physical attributes [10]. Healthcare professionals and patients who joined the trial reported that they had the skills to use the website. However, patients were not necessarily physically capable of eating and preparing food, nor consuming large numbers of calories, for example via oral nutritional supplements. Using the intervention could be increasingly challenging as patients lost functional capacity and appetite.

#### 3.5.1 Skills Needed to Use the Website

The feedback from healthcare professionals regarding the website was mixed, with some describing it as user‐friendly and facilitating the smooth delivery of the intervention, whilst others described technological challenges. For example, there is a need to constantly switch between the guidance for delivering the intervention, the website and the video consultations when conducting video consultations with patients. Nonetheless, they felt capable of using the website. One healthcare professional was concerned that not all patients had the digital literacy required to use the website, which could have affected recruitment to the trial. Healthcare professionals described how they trained patients to use the website by helping them to overcome technological barriers, and how the intervention visits were useful for this purpose. Although some healthcare professionals commented that this training was lengthy, they appreciated that it took time for patients to learn how to navigate the website. Patients in the sample described finding the website straightforward and user‐friendly: ‘*Very user friendly, very easy to navigate*’ (P13, late). They raised minor issues about the speed of the search engine in the food diary, the time commitment associated with inputting food diaries, and annoyance with the system's lack of readily available common foods and brands within the food diary.

#### 3.5.2 Poor Functional Capacity Can Make Eating and Food Preparation Challenging

Most patients interviewed experienced a slow decline in symptoms over the 12‐month trial. Patients, caregivers and healthcare professionals reported that issues with dexterity, mobility, swallowing and breathlessness affected patients' ability to shop, cook, open packaging and consume enough food to meet intervention targets. Some patients declined to participate due to difficulties not only with eating but also with tasks like weighing themselves.

Healthcare professionals noted that physical deterioration could cause anxiety for both patients and caregivers, which sometimes needed to be addressed to support continued engagement. Fatigue often worsens fine motor issues, further hindering eating. Nausea from medication, or from ALS itself, could reduce appetite, complicating efforts to meet calorie goals. In this context, calorie targets were sometimes perceived as overwhelming or unachievable.

Particularly over time, they're then getting symptoms that can challenge eating and drinking and appetite, whether it be swallowing or physical eating difficulties, or buying food or preparing food.

HP3

I'm having increasing problems conveying food to my mouth. I'm not needing to be spoon fed just yet but I think that's not that far off.

P9, early

#### 3.5.3 The Challenge of Using Oral Nutritional Supplements

Both healthcare professionals and patients discussed the importance of promoting diets with oral nutritional supplements. One patient described how useful and easy the supplements were in ensuring they met their daily calorie targets:

I don't worry at the end of the night if I haven't met my calorie intake, and just drink it.

P14, late

However, a more common view of oral nutritional supplements in this sample was that they were challenging to consume. For example, a caregiver highlighted that while the patient incorporated the supplement drinks into their diet, there was a psychological barrier that meant they did not use supplements as often as expected because they wanted to be able to eat food.

I don't think [the patient] has as many drinks as he should do, you know the supplement drinks, I think that's like a psychological thing as well, because in his head he thinks I can still eat, I want to eat, but he does take them, I just don't think he utilises them as well as he should do.

C2, late

### 3.6 Opportunity: The Need for Informal Caregiver Support

The COM‐B framework describes opportunity as the external factors that help or hinder behaviour, including time, money, a suitable environment or social support. The key issue in this study was that informal caregivers could offer social support and thereby increase the opportunities for patients to use the intervention and change their behaviour. Informal caregivers could address some of the capability challenges described earlier by supporting patients both physically and emotionally. However, participating in the research and, to some extent, the intervention could feel like a burden for some patients and informal caregivers, which could then limit opportunities to change behaviour.

#### 3.6.1 The Importance of Social Support

Solutions to problems caused by patients' declining functional capacity included informal caregivers buying and preparing food and addressing functional limitations by, for example, leaving packaged food unwrapped in the fridge. Informal caregivers also offered significant emotional support, helping patients deal with the aftermath of the diagnosis, helping make changes to life plans, and offering encouragement to meet the intervention calorie targets. Some healthcare professionals identified this shared responsibility between patient and caregiver for the intervention as a driver for success, describing informal caregivers as invested in the intervention as much as the patients.

[Patient name] gets breathless walking so far, so either my daughter or me will do the shopping now.

C5, late

Even though informal caregivers facilitated consumption of calories, some patients did not have informal caregivers but still used the intervention. One healthcare professional described a patient in the trial who did not have caregivers or a social network but who found that engagement with the intervention, and the healthcare professionals delivering the intervention, was possible and indeed had helped in alleviating their social isolation.

One of the patients that I had a visit with last week loves it, he doesn't have much of a social network, he doesn't have a support system but this definitely keeps him engaged.

HP1

#### 3.6.2 Challenges of Fitting the Intervention Into Life

Some patients described how they prioritised the intervention, adapted it to fit into their lives or adapted their lives around the intervention. That is, they created opportunities to use the intervention. Others described leading busy lives or how they were dealing with the effects of having a degenerative disease, so could find using the intervention, and being in a research project, a burden. Time constraints emerged as a recurring theme, reducing the opportunity to use the intervention and change behaviour. Some patients described how the intervention interrupted their usual daily routines and took time that they did not have. As described earlier, those with support from family and friends could address this by having support from these informal carers.

Some patients dropped out of the intervention. Healthcare professionals attributed drop‐out to the challenges posed by the research burden while trying to deal with a terminal diagnosis, which affected even those who found the intervention acceptable. Informal caregivers could also experience difficulty balancing the demands of the intervention with their work commitments and other caregiving responsibilities, reducing the opportunity to use the intervention.

It is a lot of hard work to make sure that you're hitting the calories and doing this and doing the food diaries and then obviously it adds a lot more appointments.

P&C1, early

### 3.7 Motivation: Conflicting Motivations Could Present Challenges

The COM‐B framework describes motivation as an individual's willingness to engage in the behaviour and override competing behaviours or desires. In this study, healthcare professionals, patients and informal caregivers recognised the value of the intervention. Patients were motivated to use the intervention because they believed it might slow disease progression (beliefs about consequences), it gave them the opportunity to take action in the context of a degenerative disease which has a short prognosis and no cure (optimism), and it allowed patients to practise altruism by participating in research that could drive improvements in treatments in the future (goal). However, these motivations were challenged by the desire not to become overweight, especially in the context of having spent their lives controlling their weight. Related to this was the challenge of eating unhealthy foods when they were accustomed to eating healthily. Patients reacted differently to this challenge. Some accepted it and worked hard to reach their calorie target. This calorie target was a motivating factor built into the intervention. Others adapted their calorie targets to keep them motivated.

#### 3.7.1 Belief That the Intervention Could Slow Progression of ALS

Healthcare professionals expressed varying beliefs about the intervention. Some were confident in the benefits of meeting calorie targets, while others valued the nutritional guidance but were sceptical about patient outcomes, particularly when patients were reluctant to gain weight. Concerns were also raised about promoting unhealthy foods. One healthcare professional noted that advising high‐fat, high‐sugar foods conflicted with usual guidance, so they adapted the intervention to include healthier high‐calorie options like bananas and avocados.

One professional suggested that slow recruitment may have been due to patients prioritising drug trials, perceived as more promising. Some participated in both trials, but others chose drug trials instead. Once enrolled, understanding the intervention's purpose and evidence base helped motivate patients to follow recommendations and prevent weight loss.

Patients were driven by potential health benefits and appreciated regular monitoring and feedback. Five patients, unprompted, said they would continue the high‐calorie diet post‐trial, though only one intended to keep using food diaries. One caregiver clearly understood the intervention's rationale and used it to motivate the patient.

We constantly have this conversation—‘if you were a lot thinner and you [weren't] getting those calories, I think your deterioration would be quicker’.

C3, early

#### 3.7.2 A Desire to Take Action in the Context of a Degenerative Disease

Patients described being motivated to join the trial and use the intervention because they wanted to take action themselves in the face of feeling out of control with a progressive, terminal diagnosis. They were also motivated by altruism in terms of contributing to research to help future generations slow disease progression, which contributed to patients feeling useful, giving them focus and meaning.

It gives some reassurance not just for myself, but also the people around you, family that you're trying something, you're doing something, and there seems to be reasonable evidence that this will have a positive effect.

P3, late

#### 3.7.3 Unwelcome Weight Gain

Some patients highly valued their body shape and had a history of working hard to maintain it through moderating their food intake, intermittent fasting and vigorous exercise. They therefore were concerned about, and demotivated by, how the intervention might affect their body shape, not simply in terms of gaining weight but also about weight distribution. They noted weight gain around the abdomen and some of the practical challenges associated with this, such as clothes not fitting and decreased mobility. Weight gain for a wheelchair user caused worry that they might not fit in their wheelchair. In most of these cases, there was a sense of acceptance of this and in some instances, resignation, as they traded body shape with the potential for health benefit.

I always had a flat stomach, and now I've got quite an extra spare tyre around my middle, which is a pain. But I think it's one of the least of my worries.

P9, early

While some patients celebrated when they had gained weight, others struggled with the psychological effects of this weight gain. The contrast between their previous lifestyle choices and the intervention's focus on high‐calorie foods affected their motivation to continue. However, the patients in our sample continued using the intervention because they were motivated by the belief that they could attain a desired goal of prolonged quality of life and survival. To stay motivated, some patients adapted the intervention, setting lower calorie targets for themselves or requesting no increase in targets, so they could maintain rather than gain weight.

#### 3.7.4 The Motivating Power of Target Setting

Healthcare professionals, patients and informal caregivers described the motivational benefits of setting calorie targets. Healthcare professionals highlighted how targets provided a clear structure and purpose because patients saw meeting these targets as a proactive step in managing their health. Setting and achieving calorie targets could significantly enhance motivation if they were perceived as realistic and achievable by patients, but demotivating if perceived as unattainable. One patient likened the targets to having a prescription, seeing them as a way of achieving the end goal.

if I'm putting the right amount of fuel, as much as I can, into my body to help my muscles, that's got to be a good thing … that for me is motivational because it keeps me going.

P6, early

## 4 Discussion

### 4.1 Summary of Findings

Healthcare professionals, patients and informal caregivers in this study recognised the value of the intervention and engaged with it. Patients were motivated by the belief that it might slow disease progression, but often relied on informal carers due to limited ability to prepare and consume the required high‐calorie intake. Concerns about weight gain sometimes led to adaptations, with healthcare professionals and patients reducing calorie targets.

Our findings align with previous studies of nutritional interventions in ALS [7, 8, 16]. These have similarly noted that physical limitations can restrict patient engagement, caregiver support can help overcome these barriers, and concerns about weight gain are common [7, 8].

A systematic review of the efficacy, safety and tolerability of high‐calorie diets in ALS patients published in 2023 identified only four relevant studies [6]. The conclusion was that supplementation is safe and tolerable but has not been shown to improve weight maintenance or quality of life. Thus, the trial associated with our process evaluation is a welcome contribution to the evidence base about nutritional interventions in this patient group.

### 4.2 Strengths and Limitations

The findings of this process evaluation are an important addition to the evidence base about increasing calories to improve quality of life in people with ALS. It identified how people with ALS in slow decline want to and can use a new behaviour change intervention, and how it becomes more of a struggle as their physical function deteriorates. The sample was diverse in terms of including a variety of ALS centres and the perspectives of healthcare professionals, patients and informal caregivers. However, there were some limitations. First, the measurement of fidelity largely relied on the subjective perceptions of healthcare professionals delivering the intervention regarding their adherence to the intervention protocol. Only a few intervention visits were audio recorded, so this component of the study may be susceptible to performance bias, whereby healthcare professionals may change their behaviour because they are being observed. Second, we interviewed patients who were alive and stayed in the trial for at least a few months; some patients died or deteriorated in health quickly and withdrew from the trial, often reporting that juggling a terminal diagnosis and additional research commitments was too burdensome. Third, recruitment to the trial has consistently been slower than expected, and it may be that the intervention appeals to a specific subset of individuals who are attracted to its potential and feel capable of using it. That is, the intervention may need to be seen as feasible only for a subset of patients rather than all ALS patients within 2 years of symptom occurrence. Uptake might be higher if this intervention were offered as part of routine care, as the added burden of research participation would be removed, and patients would not feel they had to choose between competing trials. Some patients also made considerable effort to speak, responding briefly or allowing caregivers to provide more detailed reflections.

### 4.3 Implications

This process evaluation reports on a new intervention for nutritional management in people with ALS, addressing a gap due to limited evidence on proactive nutritional care [5, 6, 7]. The intervention may appeal to some patients by offering a sense of hope and control in a context where autonomy often feels lost. Patients understood the rationale and were motivated to continue, even after the trial, despite physical decline, concerns about weight gain, and difficulty finding time to engage with the intervention while managing complex health needs. It appears feasible and acceptable, with potential for effectiveness.

However, it may not be suitable for all, particularly those lacking the capacity to use a computer or to prepare food. Informal caregivers play a key role, compensating for patients' physical limitations and supporting ongoing use. Developers of complex interventions in ALS should account for this in future designs.

Healthcare professionals also supported patient use of the intervention website. A key challenge was maintaining fidelity while adapting to individual needs. This intervention was designed for flexible delivery, and such tailoring appears both feasible and necessary. The intervention component, where healthcare professionals in different ALS centres met monthly with the research team, allowed for the sharing of challenges and understanding that it was acceptable to adapt the intervention to meet patient needs. This has implications if the intervention were to be used in routine practice—that training should emphasise that tailoring is necessary.

There were some implications for recruitment to future trials in ALS, although it is important to recognise that patients who declined to participate in the trial were not interviewed, and these insights were identified from healthcare professional interviews. First, the trials unit was trying to open sites during the Covid‐19 pandemic and aftermath when a number of trials failed to recruit. This is unlikely to affect future trials in ALS. Second, interventions with digital components require digital literacy, so they may exclude some patients; this is relevant to all trials of digital interventions and is not specific to ALS. Third, behaviour change interventions like this one will compete with drugs when patients are selecting which trial to participate in, and drugs may appear to patients to offer more potential for slowing progression. Fourth, intervention burden may need to be minimised to recruit and retain patients within trials of complex interventions in this population; this is something for all future trials of complex interventions in ALS to consider.

## 5 Conclusion

This intervention appeared to be feasible to deliver, delivered with fidelity, acceptable and engaging for some patients who were well enough, and had the capability or support, to use it.

## Author Contributions

**Katie Hullock:** data curation, formal analysis, investigation, writing – original draft. **Alicia O'Cathain:** methodology, writing – original draft, writing – review and editing. **Fiona Sampson:** data curation, investigation, writing – review and editing. **Jonathan Woodward:** data curation, writing – original draft, writing – review and editing. **Elizabeth Coates:** conceptualisation, funding acquisition, supervision, writing – review and editing. **Theocharis Stavroulakis:** writing – review and editing. **Sean M. White:** conceptualisation, funding acquisition, supervision, writing – review and editing. **David White:** conceptualisation, funding acquisition, writing – review and editing. **Paul Norman:** conceptualisation, funding acquisition, writing – review and editing. **Christopher McDermott:** conceptualisation, funding acquisition, supervision, writing – review and editing.

## Disclosure

The views expressed are those of the authors and not necessarily those of the NIHR or the Department of Health and Social Care.

## Ethics Statement

The study was approved by North West—Greater Manchester West Research Ethics Committee, reference: 20/NW/0334 IRAS project ID: 275949.

## Consent

All participants provided informed consent before taking part in this study. All participants also provided consent for the anonymised publication of their data. They were informed about the study's purpose, potential risks and their right to withdraw at any time. They were informed that their responses would be used in academic publications and were assured that no personally identifiable information would be disclosed.

## Conflicts of Interest

C.J.M. is supported by the NIHR Sheffield Biomedical Research Centre and an NIHR Research Professor award. The authors declare no financial conflicts of interest.

## Data Availability

The qualitative data generated and analysed during this project are not available for sharing due to ethical, legal and confidentiality constraints. In accordance with UK clinical trial regulations, GDPR, and Health Research Authority (HRA) guidelines, participant data generated from the interviews conducted contain sensitive information that cannot be fully anonymised and must be protected. As the trial is ongoing and participants are still being recruited and followed up, all research data will remain strictly confidential and will not be made available to external parties at this time.
